# Automatic gait analysis in canines using computer vision

**DOI:** 10.3389/fvets.2026.1729697

**Published:** 2026-05-05

**Authors:** Brian Phelan, Turlough Mc Nally, Laura Cuddy, Gerard Lacey

**Affiliations:** 1 Department of Electronic Engineering, Maynooth University, Maynooth, Ireland; 2 Veterinary Specialists Ireland, Summerhill, Ireland

**Keywords:** canine gait analysis, computer vision, locomotion, mesh reconstruction, pose estimation

## Abstract

Automated canine gait analysis using computer vision has the potential to extend objective canine gait assessment beyond specialized, controlled laboratories into domestic environments, but the field is comparatively less mature than human methods. This review explores the state-of-the-art for vision-based canine gait analysis, with a particular emphasis on single-camera (monocular) articulated pose and shape reconstruction, along with the extraction and interpretation of clinically relevant gait parameters. Across the literature, current pipelines reconstruct anatomical and surface representations of canines from images and video, yet rarely achieve the biomechanical fidelity or validation against gold-standard references such as motion capture, pressure walkways or fluoroscopy. Three requirements emerge from the literature: robust monocular 3D reconstruction sufficiently accurate to measure soft-tissue artifacts (approximately 10–20 mm), a standardized set of gait parameters aligned to veterinary assessment practices, and a shift from parts-based to holistic gait analysis. We highlight priority research directions to improve monitoring of canine gait in real-world settings including breed-representative datasets, synthetic training data to real-world data adaption, and ensemble learning for pathology identification. Addressing these gaps could allow for objective, longitudinal monitoring of canine gait in both veterinary practices and domestic environments. We advocate for increased interdisciplinary collaboration to foster innovation and establish new standards in the field.

## 1 Introduction

Gait analysis is a sub-field in bio-mechanics which refers to the systematic study of animal and human locomotion by measuring body movements, mechanics, and the activity of the muscles. Gait analysis quantifies complex patterns of limb movements and the forces generated at various speeds in order to gain an understanding for the underlying mechanisms and principles of locomotion. An objective investigation of both normal and pathological patterns of movement furthers the diagnoses, treatment and rehabilitation of animals and humans with conditions affecting their ability to move. The analysis of gait is typically performed in clinical environments for both animals and humans, and is usually not possible in domestic scenarios due to the expertise and equipment required. Traditional methods of gait analysis require trained operators performing various examinations such as visual and palpation-based assessments, as well as utilizing specialized kinetic laboratories to perform deeper biomechanical analysis. New technologies have the potential to scale the benefits of gait analysis for veterinarians, including enhanced post-operative care, triaging animals in home settings, and the analysis of longitudinal health data to inform the current state of an animals health. This can be further extended to cover high performance activities, sports and service work.

While there has been significant work for computer vision methods in humans, its application to animals remains comparatively underdeveloped despite their long-treasured role in society. Canines represent one of the most clinically evaluated companion animal and also exhibit a wide variation in morphologies across the world. Lastly, the increasing availability of household videos has created new opportunities for enhanced domestic monitoring of canine health and mobility. Together, these factors justify a focused review, as developments for dogs cannot necessarily be generalized from other quadrupeds, but may introduce opportunities for such generalization. Therefore, the purpose of this review is to investigate and synthesize computer vision-based gait analysis approaches in canines. Specifically, current approaches in articulated pose and shape reconstruction, clinically relevant gait parameter extraction and locomotion analysis are examined. By consolidating these studies, the review aims to clarify the state of the field, identify gaps, and outline future research opportunities.

### 1.1 Review methods

**Figure 1:**
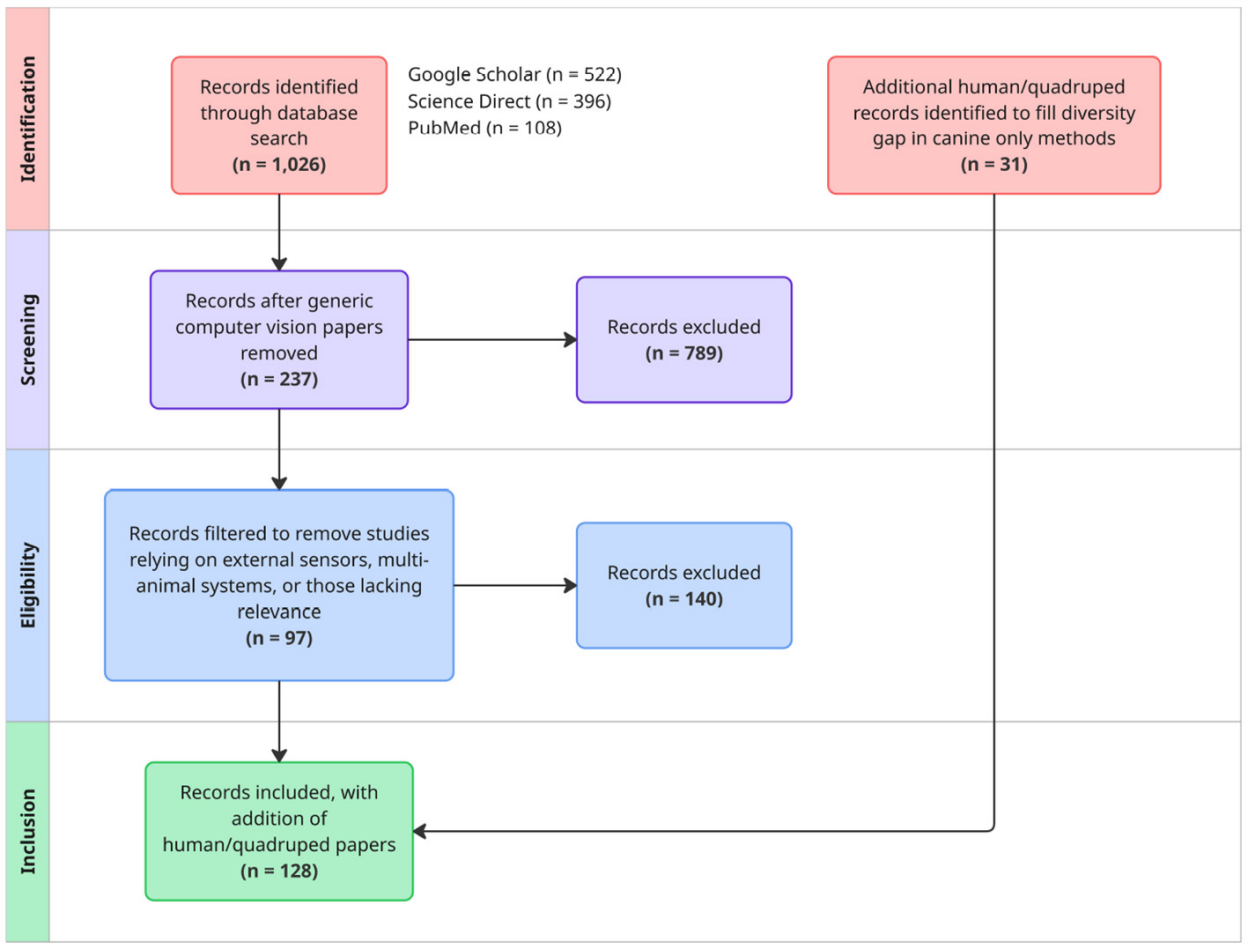
PRIMSA-style flow diagram of review methods and study selection.

A comprehensive search was conducted for this narrative-style review in Google Scholar, Science Direct and PubMed databases, covering literature up to October 2025, as seen in Figure 1. Search terms included: “computer vision,” “canine,” “dog,” “gait analysis,” “gait parameter extraction,” “pose estimation,” and “mesh reconstruction.” Search terms were combined using boolean operators and included:

- (“computer vision”) AND (“canine” OR “dog”)
- (“gait analysis” OR “gait parameter extraction”)
- (“pose estimation” OR “mesh reconstruction”)

#### 1.1.1 Inclusion criteria

Studies were included if they met all of the following criteria:

- Computer vision, image processing, or video-based gait analysis relevant to canines.
- Methods for 2D or 3D pose estimation, mesh reconstruction, or gait parameter extraction.
- Contributions to the methodological understanding of automated gait analysis.
- Published in peer-reviewed journals or conferences.
- Contained enough technical detail to be meaningfully evaluated.

#### 1.1.2 Exclusion criteria

Studies were excluded if they:

- Focused primarily on wearable sensors or force/pressure-based systems without vision, except for when providing context to traditional methods.
- Presented generic computer vision techniques.
- Were outdated studies (more than 10 years old) unless they presented a foundational methodology still widely referenced or applied.
- Used multi-animal tracking, robot motion, or computer graphics unrelated to gait analysis.

#### 1.1.3 Screening procedure

The initial search returned 1,026 publications: Google Scholar (522), ScienceDirect (396), and PubMed (108). After removing non-relevant records and studies which focused primarily on generic computer vision papers, 237 studies remained. Further filtering excluded studies relying on external sensors, multi-animal systems, or those lacking relevance. This resulted in 97 core canine computer vision studies. Due a lack of diversity in the techniques used with canines, some human/quadruped gait analysis and pose estimation studies were included to ensure adequate representation of state-of-the-art methods in articulated shape or scene reconstruction. This resulted in the addition of 31 cited studies. After screening and applying the above criteria, 128 studies remained for detailed consideration in this review.

For each included publication, the following details were extracted:

- Computer vision method.
- Dataset characteristics (real, synthetic, breed diversity).
- Architecture/Backbone.
- Extracted gait parameters and associated methods.
- Evaluation metrics, where applicable.
- Clinical relevance or application.

The studies were grouped into (1) Articulated Pose and Shape Reconstruction, and (2) Gait Analysis. Throughout this review, studies are assessed for methodological quality, including validation against gold-standard measurements, reporting of quantitative accuracy, breed diversity, and clinical relevance, to clarify the strength and limitations of the evidence.

### 1.2 Review structure

To provide context to the emergence of computer-vision methods in gait research, Section 1.3 begins by briefly discussing the history of locomotive analysis in humans and animals. Building on this foundation, Section 1.4 examines the traditional techniques used in canine gait analysis. While well-established, these methods reveal important limitations such as subjectivity, accessibility, and dependence on specialized equipment. Together, they motivate the shift toward automated, vision-based systems. Ultimately any such system must evaluated against clinical standards such as Clinical Metrology Instruments (CMIs) and commonly used gait parameters, which this section also introduces. Sections 1.5.1, 1.5.2 introduce the core concepts for computer vision approaches, asking the question: how can gait information traditionally obtained through complex systems and markers be replicated or improved using only visual data? To answer this, we require an understanding of pose estimation as a foundation upon which all possible automated gait analysis methods depend. Section 2.1 transitions deeper into to the vision-based approaches, organized into Articulated Pose & Shape Reconstruction (Section 2.1) and Gait Analysis (Section 2.2). This structure mirrors the pipeline used in automated systems, from firstly retrieving the dog's form and structure, then quantifying its movement, and lastly deriving meaningful biomechanical insights. Within each of their sub-sections, we compare the methodologies, highlight recurring obstacles, and show gaps that constrain real-world applicability. Section 3 compares findings from Sections 1.4, 2, showing convergent requirements across traditional and computer vision approaches. Lastly, Section 4 interprets the implications of these findings for automated canine gait analysis, focusing on the why these gaps matter and what must be addressed for these methods to become clinical reliable. Lastly, we outline the major barriers limiting clinical deployment, as well as the broader significance of computer vision in canine and animal research.

### 1.3 Background

The measure of musculoskeletal function was first documented by Aristotle in *De Motu Animalium* (1), with no documented further developments until Giovanni Borelli's updated version in the 17th century (2). Aristotle analyzed the different movements of animals, but from a broad biological and metaphysical perspective. Giovanni Borelli, often referred to as the “Father of Biomechanics,” applied a more mathematical and mechanical approach, focusing on the expanded musculoskeletal understanding of his time. Early on, it was discerned that the gait of animals or humans was defined by the biomechanics of locomotion, which was divided into kinematics and kinetics. Muybridge's groundbreaking photographic sequences in the late 1800s showed the sequential phases of animal and human movement, debunking previously held beliefs, such as the airborne phase of a horse's gallop (3). At the same time, Braune and Fischer's kinematic investigations revealed the sinusoidal trajectory of the body's center of mass during unassisted walking, giving a possible inheritance to mechanical conservation of energy (4). Alexander's studies spanned various terrains and speeds and showed the evolution of specialized gaits and limb configurations in animals to optimize energy use and agility in their natural habitats (5). Collectively, these foundational observations have enriched our understanding of animal gait, emphasizing the confluence of evolution, biomechanics, and environmental adaptation.

### 1.4 Traditional canine gait analysis

#### 1.4.1 Visual based observations

Visual-based observations of gait in dogs is a subjective method whereby findings from an evaluation by a trained operator are semi-quantified using a form of numerical rating scale. These scales include: numerical lameness grading scales (0–4 or 0–5), such as the Visual Analog Scale (VAS), which has been used in human as well as veterinary studies (6), and owner or handler questionnaires such as the Canine Brief Pain Inventory (CBPI) or Liverpool Osteoarthritis in Dogs (LOAD) (see Section 1.4.6). The main shortcoming of visual-based observations is that they are subjective and highly operator-dependent, requiring expert knowledge to effectively use. Even when performed by experts, subjective gait analysis has been shown to be highly inaccurate (7).

#### 1.4.2 Kinematic motion capture

Motion capture systems track the trajectory of reflective markers during locomotion, providing a 3D quantitative assessment of gait kinematics, including joint angles, limb trajectories and spatiotemporal gait parameters. Markers are typically placed at key anatomical locations along the dog's body, for example the iliac crest and greater trochanter (9). The resultant objective kinematic data is highly beneficial for evaluating gait and detecting pathologies, as seen in studies such as Hamilton et al. (10). However, the attachment of the markers to the relatively loose skin and hair of dogs can introduce a shift of location relative to the underlying bone and the haired areas may need to be shaved in order to ensure accuracy during specific movements (11). As a result of these limitations, false positives and inaccurate data may be obtained, as shown by Lin et al. (11) through their consistent underestimation of flexion/extension of adduction/internal rotation when the stifle was flexed beyond 90°. The use of kinematic motion capture is largely limited to research institutions due to the equipment, time and expertise required to operate and interpret clinically-relevant data.

#### 1.4.3 Pressure sensitive walkways or force plates

**Figure 2:**
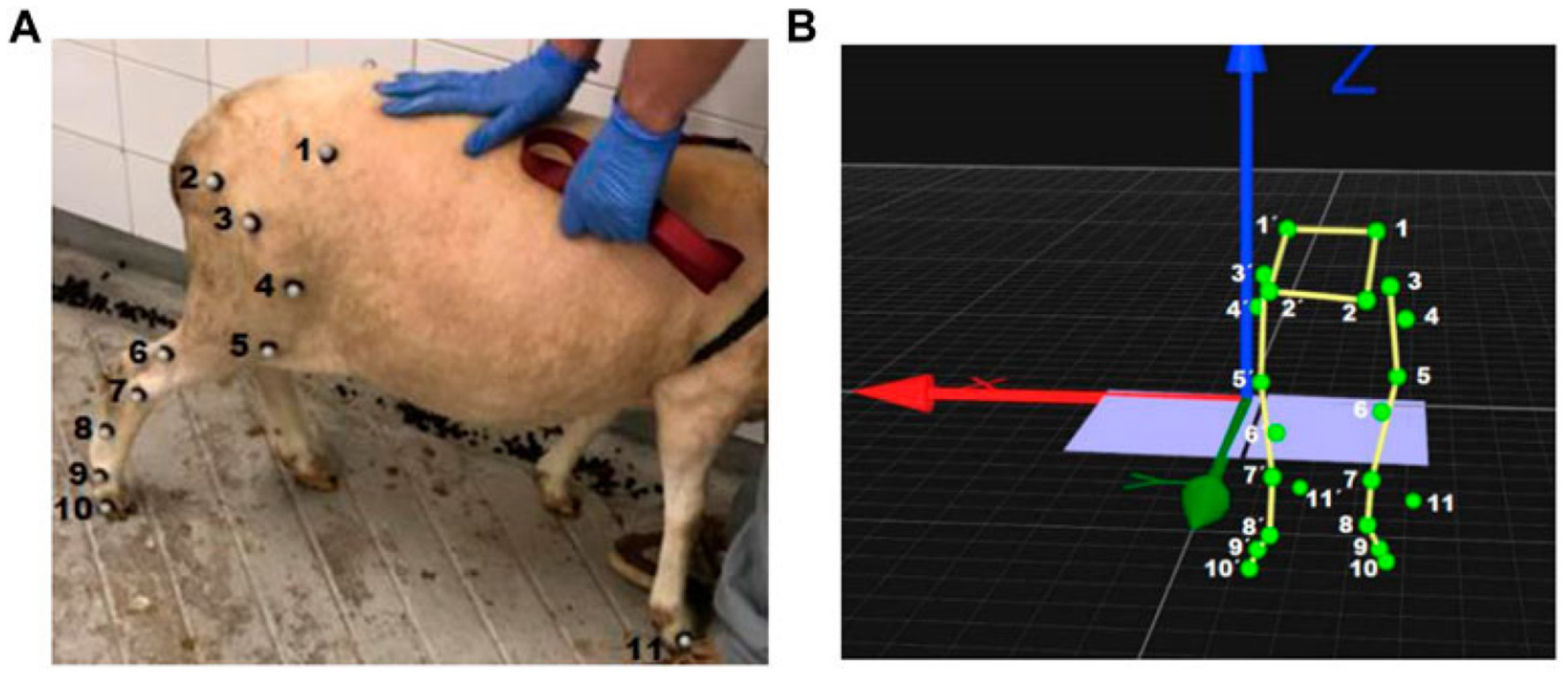
3D visualization of retro-reflective markers placed on key anatomical landmarks of sheep from Silve et al. (77), licensed under CC BY 4.0. **(A)** shows the markers being placed on the right hind legs of the sheep, and **(B)** shows the visualization of the markers inside 3D software.

Force plates are the gold standard for detailed biomechanical data, however, are challenging and time-consuming to use. The use of force plates has largely been supplanted by pressure sensitive walkway systems (12) (as seen in Figure 2), or pressure mats due to their availability and ease of use in a clinical settings (13). Pressure mats are embedded with thousands of sensors that record how the paws contact the ground during walk or trot. Pressure mats capture both temporal (timing) and spatial (force/pressure distribution) aspects of locomotion, presenting them in a format that can readily be interpreted by a clinician. The output of pressure mats includes temporal parameters (stance time, swing time, stride time), spatial/force parameters (peak vertical force, pressure distribution within the paw, center of pressure movement), and symmetry indices (compare right versus and left and fore versus hindlimbs). Metrics derived from the pressure walkway systems, provide a comprehensive understanding of gait dynamics, aiding in diagnostics, therapeutic decisions, and rehabilitative measures. This can be seen by Assies et al. (14) through evaluating the effects of an undenatured collagen type-2-based nutraceutical on Recovery Time after TPLO in dogs, using objective gait analysis as the primary outcome measure.

#### 1.4.4 Treadmill analysis

Treadmill analysis ensures clinicians can achieve consistent and repeatable gait cycles for analysis. This method offers a controlled environment for data acquisition, mitigating the inconsistencies typically seen in overground locomotion (9). Furthermore, the stationary nature of treadmill walking permits continuous data collection, which might be challenging with conventional overground methods. The ability to adjust treadmill speed and incline also allows clinicians to examine gait under different conditions and stressors, therefore providing a comprehensive understanding of its gait via parameters such as kinematics (15) and kinetics (16). Limitations to treadmill analysis including the requirement for acclimatization, as well as potential altered gait mechanics on treadmill versus overground.

#### 1.4.5 Wearable sensors

In the contemporary landscape of canine gait analysis, wearable sensors have carved a niche for themselves, heralded for their real-time data acquisition capabilities and potential for extended monitoring. These sensors, predominantly Inertial Measurement Units (IMUs) and accelerometers, are designed to capture intricate details of motion and orientation, facilitating an in-depth evaluation of a canine's gait (17). IMUs, an amalgamation of accelerometers, gyroscopes, and occasionally magnetometers, measure a spectrum of parameters from linear accelerations and angular velocities to magnetic fields (18). This multifaceted data acquisition enables the calculation of a body's orientation and movement in a 3D framework (19). Accelerometers, often deployed independently of IMUs, are pivotal for assessing lameness, as they provide granular data on the intensity and trajectory of movement (18). Their compact design and non-intrusive nature make wearable sensors an invaluable asset for prolonged, continuous monitoring, be it in a clinical or domestic setting.

#### 1.4.6 Clinical metrology instruments

Clinical metrology instruments (CMIs) are standardized, owner-reported questionnaires designed to quantify health-related domains such as pain and mobility in canines. This form of structured feedback allows the owner to observe their dogs in a domestic setting, where the animal is at its most comfortable, eliminating common behaviors like stress that often skew results of observations. By translating the varying dimensions captured into a numerical scoring system, CMIs enable both the monitoring and evaluation of interventions and illnesses such as osteoarthritis, which then typically need to be confirmed with diagnostic imaging. An overview of the most commonly used CMIs, adapted from Gruen et al. (20), is presented in Table 1. Implementations of these CMIs vary, and are often given to veterinarians in conjunction with home video taken by the owner, as discussed by Gruen et al. (20). These videos are typically not of the required quality due to a variety of factors such as: portions of the video not containing the animal, unsteady camera, and a lack of specific movements that help accentuate specific symptoms.

**Table 1:** Clinical metrology instruments, adapted from Gruen et al. (20).

Tool	User	Validity	Acute/chronic
Canine osteoarthritis staging tool (COAST) (94)	Owner, veterinarian	Not validated	Chronic, osteoarthritis
Liverpool osteoarthritis in dogs (LOAD) (87)	Owner	Validated	Chronic, osteoarthritis
Canine brief pain inventory (CBPI) (95)	Owner	Validated	Chronic, osteoarthritis
Helsinki chronic pain index (96)	Owner	Limited validation	Chronic, osteoarthritis

#### 1.4.7 Gait parameters

The parameters listed in Table 2 are predominately kinematic and spatiotemporal measures extracted from the literature. They comprehensively capture limb and body movements, joint excursions, and spatiotemporal aspects of canine gait. They do not include kinetic measures such as ground reaction forces, joint moments, etc.

**Table 2:** Comprehensive overview of key kinematic and spatiotemporal parameters used in canine gait analysis, presented with concise definitions and indications of whether each parameter can be readily obtained from 2D/3D imaging.

Parameter	Definition	2D	3D
Displacement	Linear change in position of a marker (e.g., on a limb) over time.	Possible (sagittal plane only)	Required for full spatial measurement
Distance	Total path length traversed by a point during a gait cycle.	Approximate in 2D (planar path)	Required for true 3D trajectory length
Joint angles	Angle between two adjacent limb segments measured at a specific joint.	Possible (sagittal plane components)	Required for multi-planar angles
Angular velocity	Rate of change of a joint angle over time.	Computable if angle obtained in 2D	Required for full 3D rotational velocity
Range of motion	Difference between maximum and minimum joint angles during a gait cycle.	Measurable in sagittal plane only	Required for multi-plane ROM
Joint trajectories	Path traced by a joint (marker) in space over time.	Planar trajectory only	Required for full trajectory
Pelvic drop	Vertical excursion of the pelvis relative to a horizontal reference during stance.	Detectable in sagittal 2D	Required for frontal-plane obliquity
Center of gravity	Spatial location of the whole-body center of gravity during gait.	Not accurate in 2D (lateral shifts lost)	Required for accurate COG estimation
Gait cycle	Time interval from initial contact of one limb to the next contact of that same limb.	Measurable from 2D foot-fall events	3D not required for timing
Stride time	Duration of one gait cycle (seconds).	Measurable in 2D via frame timing	3D not required
Step time	Time between initial contact of one limb and initial contact of the contralateral limb.	Measurable in 2D if foot-falls visible	3D not required
Swing time	Time during which a limb is not in contact with the ground.	Measurable in 2D using foot-off/on events	3D not required
Stance time	Time during which a limb is in contact with the ground.	Measurable in 2D using foot-on/off events	3D not required
Step length	Fore-aft distance between successive paw placements of contralateral limbs.	Approximate in sagittal 2D if planar	Requires 3D for off-plane motion
Stride length	Fore-aft distance between successive paw placements of the same limb.	Approximate in sagittal 2D if planar	Requires 3D for off-plane motion
Step width	Lateral distance between placement of contralateral paws.	Not measurable in sagittal 2D	Requires 3D or frontal/dorsal view
Gait symmetry	Ratio or difference between right and left limb parameters (e.g., timing).	Computable from 2D spatial/temporal data	3D not required for symmetry timing
Single support time	Duration during which only one limb is in contact with the ground.	Measurable in 2D if foot-fall events visible	3D not required
Double support time	Duration during which two limbs are simultaneously in contact with the ground.	Measurable in 2D if overlapping contacts visible	3D not required
Cadence	Number of steps taken per minute.	Derivable from 2D step count and time	3D not required

These parameters were gathered across the literature, including Pálya et al. (97), Lin et al. (28), Miqueleto et al. (98), and McLaughlin (99).

### 1.5 Computer vision-based gait analysis

Gait Analysis through computer vision can be broken down into three key building blocks:

- **Articulated shape and pose reconstruction (2D and 3D):** to create an objective representation of the dog's body configuration, either as a set of anatomical keypoints (skeletal pose) or full 3D mesh.
- **Gait parameter extraction:** to convert raw pose sequences into meaningful, quantifiable descriptors of locomotion that align with veterinary biomechanics.
- **Gait interpretation:** to use the extracted gait parameters and/or full motion sequence to analyse gait quality, detect abnormalities, and support clinical decision-making.

To maintain brevity, gait parameter extraction and gait interpretation are considered together under the broader term gait analysis.

#### 1.5.1 2D vs. 3D pose estimation

2D Pose Estimation aims to predict the *x, y* coordinates of keypoints (body joints) of an animal in an image or sequence of images. Typically this can be divided into frame-by-frame estimation and video-based estimation over time. 3D Animal Pose Estimation involves predicting joint positions of an animal in 3D space using one or multiple images. While 2D pose has shown great promise, it lacks much of the actionable data we need for gait analysis, as shown by Fischer (21). For the purposes of this review, we will focus on monocular pose estimation in both 2D and 3D, while only briefly touching on multi-view methods.

#### 1.5.2 Human pose estimation

Human pose estimation has functioned as the backbone of modern animal methods for a number of years. Some of the most popular models such as BlazePose (22) (3D), OpenPose (23) (2D), and PoseNet (24) (2D), have been pivotal in the development of monocular gait analysis systems i.e., Zambrano et al. (25). An overview of representative methods for human pose and shape estimation can be seen in Table 3. Unlike human pose, applying these models either directly or through naive approaches to canines presents significant challenges. This is primarily due to the substantial anatomical and morphological differences between humans and dogs, such as the number of limbs in contact with the ground, the range of motion in joints, and the variety of body shapes and sizes across dog breeds. While transfer-learning is possible in theory, it would require substantial canine-specific training data, alongside significant changes to the model's architecture. Through our research, we had made brief efforts for both direct and naive approaches in adopting the model, but achieved in poor results. In conclusion, it is fair to say that such modifications to the model with respect to canines would not be very feasible.

**Table 3:** Summary of key publications on human pose estimation.

Publication	Research direction/highlights	Data classes and training types
Hewitt et al. (100)	Fuses multi-view RGB into a neural implicit surface and jointly optimizes skeleton and per-vertex deformations, removing the need for markers and calibration through learned appearance priors.	Human; Synthetic RGB images and 3D mesh annotations from parametric models.
Rempe et al. (101)	Combines a conditional VAE motion prior with per-frame pose regression to decouple dynamics and robustly recover full-body motion under occlusion.	Human; 3D motion capture sequences (e.g., AMASS) and SMPL pose parameters.
Bazarevsky et al. (22)	Implements a two-stage, quantized CNN pipeline (person detector + 33-landmark regressor) optimized for real-time, on-device inference.	Human; RGB images and 2D keypoint annotations for body landmarks.
Güler et al. (102)	Extends R-CNN to regress dense UV surface coordinates and part labels against an SMPL template using a bespoke COCO-DensePose dataset.	Human; RGB images and dense UV coordinates mapped to the SMPL body surface.
Loper et al. (103)	Learns pose- and shape-dependent blend shapes via PCA on 3D scans to create a fully differentiable SMPL mesh model.	Human; 3D body scans, motion capture, and parametric mesh (SMPL) labels.

This table provides a structured overview of representative methods for human pose and shape estimation. These entries represent the key issues which limit 3D canine pose estimation matching the accuracy and performance of human methods, such as occlusion, multi-camera triangulation, and neural fields, pose-priors and motion capture training data. Each entry includes the research focus, target subject class, and the types of training data used. The comparison highlights diversities in model architecture, input modalities, and supervision dimensionality across methods.

#### 1.5.3 Clinical validation

For clinical applications, vision-based approaches must ultimately yield biomechanically accurate descriptors, demonstrate robustness across the morphological variability of canine breeds, and support quantitative measures that align with established clinical gait parameters. As such, in addition to surveying state-of-the-art methods in articulated pose estimation, mesh reconstruction, and motion analysis, this review considers these techniques in light of their potential for producing anatomically interpretable outputs, their amenability to validation against gold-standard biomechanical measurements, and their suitability for deployment in realistic clinical and domestic environments. This provides a unifying context for the subsequent technical methods while preserving the primary focus of the review on the methodologies of computer vision-based canine gait analysis. From a technical standpoint, agreement with gold standard measurements is key. Reconstruction accuracy is commonly reported using metrics such as Mean Per-Joint Position Error (MPJPE) for biomechanical fidelity in motion tracking, alongside more gait oriented measurements such as agreement with kinetic and spatiotemporal measurements in methods such as pressure mats.

## 2 Computer vision-based methods for canine gait analysis

### 2.1 Articulated shape and pose reconstruction

Automatic canine gait analysis requires an in-depth view of the subject's locomotion. In clinical environments, this is currently accomplished by the tracking of reflective markers placed on key landmarks of the subject (26). Pose estimation may approximate the same result, without the need for intrusive measures or clinical expertise. Table 4 provides a summary of key publications on articulated pose and shape reconstruction since 2018, reviewing representative and influential works in the literature. Computer vision-based pose estimation is typically divided into Skeletal and Mesh methods, producing different actionable parameters as a result. Skeletal methods typically train on annotated key points of joints from image frames to predict the same joint mapping on an input, whereas mesh methods typically focus on reconstructing a 3D representation of the subject.

**Table 4:** Summary of key publications on articulated pose and shape reconstruction since 2018.

Publication	Class	Training data types	2D/3D	Highlights
Zhong et al. (60)	Dog	Video, masks	3D	Provides a hierarchical, coarse-to-fine alignment framework to remove need for manual annotation of key points. Achieves faster convergence and higher fidelity than prior baselines by combining dense visual features with automatic part/temporal cues.
Lyu et al. (57, 58)	Animal	Synthetic Images, SMAL Labels	3D	Animal family-aware transformer to jointly predict pose and shape in 3D by fusing 3D labeled synthetic data with 2D key points, achieving state-of-the-art cross-species performance.
Wu et al. (104)	Dynamic scenes	Monocular Video	3D	Introduces 4D Gaussian splatting to render dynamic scenes in real time by extending static Gaussian splats into the temporal domain for continuous, interactive playback.
Shooter et al. (49)	Canines	Synthetic images, Keypoints	Both	Synthesizes photo-realistic canine images with precise 3D pose labels from a synthetic pipeline to train single-view estimators under controlled variability.
Xiao et al. (105)	Dynamic scenes	Video, Depth, Keypoints	3D	Tracks arbitrary 2D pixels in 3D by combining neural reprojection with multi-view geometry, generalizing beyond traditional keypoint methods.
Oquab et al. (106)	Dynamic scenes	Images	2D	Learns robust, label-free visual features through DINOv2's distilled-free self-supervised objective, achieving strong zero-shot performance without annotations.
Rüegg et al. (56)	Canines	Images, synthetic images, 2D & 3D keypoints	3D	Goes beyond generic shape priors by directly regressing 3D canine poses via implicit shape representations trained with silhouette and keypoint supervision.
Xu et al. (36)	Humans, quadrupeds	2D keypoints	2D	Adapts vision transformers for generic body pose by integrating modality-specific tokens and efficient sampling to handle diverse articulated configurations.
Tan et al. (63)	Humans, cats, canines	Video, NeRFs	3D	Distills articulated neural fields into a lightweight real-time model by transferring volumetric deformation priors onto compact mesh representations.
Song et al. (64)	Humans, cats, canines	Monocular & Stereo Video	3D	Reconstructs deformable scenes for embodied view synthesis by coupling dynamic neural radiance fields with dense tracking of object interactions.
Yang et al. (66)	Humans, cats, canines	Monocular Video, Skeletons, 3D Background Models	3D	Reconstructs animatable categories from monocular videos by learning category-level neural fields that enable cross-instance articulation.
Yang et al. (65)	Humans, cats, canines	Video, keypoints	3D	Builds animatable 3D neural models from casual videos by jointly registering multiple captures to a shared template with bundle adjustment and deformation nets.
Rüegg et al. (47)	Canines	Keypoints, breed labels, synthetic 3D models	3D	Leverages discrete breed embeddings as shape priors to regress accurate 3D canine meshes from single RGB images.
Li and Lee (50)	Quadrupeds	Synthetic & real images, Psuedo Labels	2D	Adopts adversarial unsupervised domain adaptation to align synthetic and real animal pose features without any labeled real-world data.
Biggs et al. (46)	Canines	Images, keypoints, 3D shape priors	3D	Introduces domain randomization on synthetic canine models to evaluate and improve cross-domain generalization of pose estimators.
Zhang and Park (107)	Quadrupeds	Multi-view images	2D	Leverages multiview registration constraints to supervise 3D animal pose models in the absence of explicit 3D annotations.
Mu et al. (32)	Quadrupeds	Synthetic images & real images	2D	Trains pose estimators solely on synthetic animal datasets and employs style transfer to bridge the synthetic-to-real domain gap.
Cao et al. (108)	Quadrupeds	Animal & human pose datasets	2D	Implements feature-level adversarial adaptation to transfer pose estimators trained on synthetic animals to real-world images.
Mathis et al. (38)	Quadrupeds	Keypoints	2D	Pioneers markerless animal keypoint tracking by adapting DeeperCut architectures to require minimal manual annotations.
Biggs et al. (54)	Quadrupeds	Video, keypoints, SMAL model	3D	Combines SMAL-based priors with video-driven bundle adjustment to recover 3D shape and motion across diverse animal species.

This table reviews representative and influential works that address pose and shape estimation in dogs, with broader comparisons to methods applied to quadrupeds, humans, and dynamic scenes. Each entry is characterized by the targeted subject class, types of training data used (e.g., synthetic images, motion capture, and monocular video), the dimensionality of supervision (2D or 3D), and a concise description of methodological innovations. The aim is to provide a structured comparison of the most recent and highly cited approaches that contribute to the development of 3D canine pose estimation, in a non-exhaustive manner. We exclude any publications that focus solely on humans or multi-class data that don't include canines, with the exception of dynamic scenes.

As these methods act as a surrogate for marker-based systems, a certain level of geometric accuracy is required for the pose to be biomechanically meaningful. There is an inherent error between marker-based systems and ground truth via fluoroscopy, largely due to the effect of skin movement artifacts. Benoit et al. (27) reported ~10–20 mm joint-center error from markers while still retaining interpretable kinematics which are widely used clinically. This is further validated by Lin et al. (11) who quantified femoral soft-tissue artifact with rms displacements of ~8–9 mm and peak-to-peak values up to ~27 mm. More recent fluoroscopy-validated multibody kinematics studies (28) further demonstrate that residual pose errors below approximately 10 mm represent high-fidelity agreement with skeletal motion, whereas larger errors approach the intrinsic uncertainty limits imposed by skin motion. Based on reported in-vivo soft-tissue artifact magnitudes in canine hindlimb motion (Root Mean Square ≈6–10 mm, peak ≈ 20–30 mm) and fluoroscopy-validated multibody optimization studies, pose errors below ~10 mm can be considered high-accuracy relative to skeletal ground truth, errors in the 10–20 mm range comparable to conventional marker-based kinematics and still biomechanically interpretable, while errors exceeding ~25 mm approach the magnitude of skin-bone motion itself and indicate loss of anatomical fidelity.

#### 2.1.1 Datasets

As seen in Table 5, existing canine pose datasets span a wide range of modalities and annotation types. From the previously discussed MPJPE limits (Section 2), we can extrapolate a set of minimum biomechanical and clinical validation requirements. Firstly, anatomical data must be derived from marker-based motion capture, fluoroscopy-validated multibody models, or anatomically rigged 3D meshes, ensuring that the joint centers are accurately represented for validation, and that associated localization errors are comparable to or below the intrinsic soft-tissue artifact bounds required for kinematic validity. Secondly, these datasets must represent the varying morphology of breeds, as parameters such as bone lengths, joint alignment and limb proportions change significantly across breeds and directly affect kinematic metrics.

**Table 5:** Summary of key datasets used in canine pose estimation.

Dataset	Modality	Size	Data types
CtrlAni3D (57)	Diffusion-generated RGB images	41,300 images	3D SMAL Mesh, 2D keypoints.
SyDog (29)	Synthetic RGB images	32,000 images	2D keypoints, bounding boxes.
RGBT-Dog (30)	RGB images (real + synthetic)	50,000 synthetic images	2D/3D keypoints, depth maps, textures, 3D models.
Animal3D (109)	RGB images, 3D SMAL parameters	3,379 images	2D/3D keypoints, SMAL parameters, segmentation masks.
Common pets in 3D (CoP3D) (35)	Smartphone RGB videos	~4,200 distinct pet videos	Object masks, camera parameters, time stamps, camera tracking quality scores.
AP-10K (70)	RGB images	10,015 labeled images, ~50,000 unlabelled images	2D keypoints, bounding boxes.
Learning from synthetic animals (32)	Synthetic RGB images, 3D models	10,000 canines	2D/3D keypoints, segmentation masks, depth maps.
RGBD-dog (31)	RGBD Video (Synthetic + Real)	830,000 synthetic	Real RGBD + motion capture, synthetic RGBD images + binary silhouette masks.
Animal-pose (110)	RGB images	~6,000 labeled, ~4,000 unlabelled	2D keypoints, bounding boxes.
Stanford dogs (34)	RGB images	10,580 images	2D keypoints, silhouette.

This table provides a non-exhaustive overview of influential datasets used in literature, including both real and synthetic data. Each dataset is characterized by modality, scale, and annotation types. Datasets were prioritized based on availability and impact/adoption across the literature. Key benchmark datasets are included among this list, excluding generic object datasets such as COCO and ImageNet.

Datasets such as SyDog (29), RGBT-Dog (30), RGBD-Dog (31), and Learning from Synthetic Animals (32), use 2D/3D annotations from real and synthetic images to counteract the lack of diverse datasets in the literature. These keypoints are a combination of manual annotation and ground-truth motion capture (31), enabling quantitative evaluation of joint localization error (e.g., MPJPE, joint angle RMSE) relative to biomechanical gold standards. Datasets such as these provide a strong foundation for both training and validation, but currently lack sufficient morphological diversity to allow models to generalize across breeds and body types, limiting their suitability for assessing inter-breed robustness and clinically relevant gait parameters. Certain dogs like greyhounds, being of dolichocephalic morphology, have longer inter-joint distances around the head, alongside thinner coats, longer limbs, and lower body fat. In this case, models trained on datasets containing these morphologies may perform better than those with longer coated dogs due to a lack of coat-based occlusion, particularly when considered in areas such as pose estimation. It is key that inter-breed variation is present, but balanced within a dataset to ensure generalization in a model. Other datasets such as COCO (33) and Stanford Dogs (34) do not provide 3D pose or biomechanical ground truth, but offer a high degree of inter-breed visual and morphological variation, making them valuable for generalization but insufficient for kinematic validation or clinical benchmarking.

The lack of biomechanical and clinical datasets introduces a key issue in validating system outputs for clinical relevance. This is further exacerbated when we consider that clinical data is not single-frame based, but rather temporally structured. This temporality is vital for understanding the gait of a canine, and as such introduces a secondary tier of validation. Datasets such as Common Pets in 3D (CoP3D) (35) provide a temporal in-the-wild dataset which would prove invaluable for studying gait, particularly in the context of automatic gait analysis. Unfortunately, this data remains anatomically unlabelled, and even with manual labeling would not yield joint centers comparable with marker-based motion tracking or fluoroscopy systems.

#### 2.1.2 Pose estimation

Current state-of-the-art models such as ViTPose++ (36), a transformer-based model, are specifically designed for 2D pose estimation, offering top-tier accuracy on benchmarks like COCO (33) for both humans and animals. ViTPose++ uses ViTAEv2 (37), an advanced pure vision transformer backbone that enhances feature extraction through stronger inductive biases and deformable token mechanisms. Unlike previous models such as BlazePose, ViTPose focuses on accuracy rather than speed and lightweight deployment, achieving an average precision (AP) of 79.4 on the COCO dataset and 76.8 on AP10K. This average precision, measuring detection and localization accuracy, does not provide direct validation to real world units and therefore cannot be assessed under the acceptable bounds of ~10–20 mm for joint-center localization, but does show the increasing quality of the state-of-the-art. In clinical gait analysis, these models still require metric scale and validation against biomechanical references. Both COCO and AP10K contain a high degree of inter-breed variability but also contain over 50 species (COCO), allowing these models be generalizable against different animals, not just breeds.

**Figure 3:**
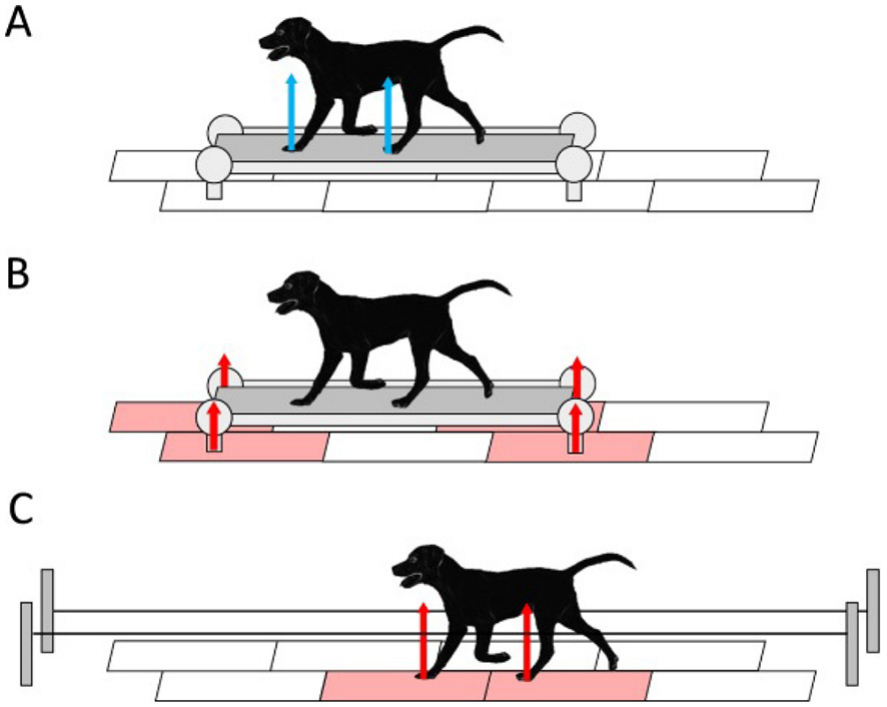
Schematic of experimental set up used by Sohnel et al. (111), licensed under CC BY 4.0. **(A)** Instrumented treadmill locomotion, **(B)** treadmill on force plates, **(C)** overground force plates. Red arrows represent the vertical ground reaction forces captured by the force plates, with the force plates colored red. Blue arrows represent the vertical ground reaction forces captured by the instrumented treadmill.

Specific to animals and humans, DeepLabCut focuses on markerless pose estimation across species (38), including canines. In this work Mathis et al. leverage transfer learning, where a deep neural network pre-trained on a large dataset (like ImageNet for object recognition), is fine-tuned for the specific task of pose estimation. The model achieves labeling accuracy comparable to that of human annotators with as little as ~200 labeled frames for training. The models ability for generalization lends itself to cross-species pose estimation, proven by their use with mice during odor-guided navigation tasks, fruit flies in a 3D chamber, and most relevantly its application to canines, through “*Predicting Dog Emotions Based on Posture Analysis”* (39), as illustrated in Figure 3. DeepLabCut also offers a 3D pose model, but requires a multi-camera system for triangulation. As is common in pose studies, these examples do not represent gait applicability and some do not provide specific accuracy measures such as MPJPE. However, Sosa et al. (40) used DeepLabCut with mice and reported an MPJPE of 8.5 pixels when compared with ground truth. While we cannot derive the error in terms of millimeters for comparison with our joint-center localization bounds, it does however suggest good within-dataset localization performance. The SuperAnimal models (41), used by DeepLabCut, utilize the Quadruped-80K dataset (42) which contains various combined sources such as Stanford Dog and AP10K. As a result, this enables strong generalizability across both animals and breeds. Despite the requirement for monocular 3D pose estimation, there are some applications for 2D keypoints such as stride time, stride length, etc., which can utilize custom models such as YOLO Pose (43) to extract keypoints based on datasets like Stanford Dogs (34, 44).

Monocular 3D pose estimation currently presents significant challenges. The scarcity of 3D annotated data is a large obstacle. As one 2D pose can correspond to multiple possible 3D poses, accurate depth estimation is required to remove ambiguity. Consequently the use of geometric prior knowledge of the target becomes a strong constraint. An example of this is the SMAL model (45) which provides a geometric prior and generalizes to many types of animals. The process is simplified by fitting the pose, shape and camera parameters of a parametric mesh, retrieving the keypoints from the vertices of the SMAL mesh on reconstruction. Skinned Multi-Breed Linear Model for Dogs (SMBLD) (46) proposed an end-to-end neural network that allowed for a wide variety of dog breeds by enriching the shape parameters of SMAL and learning additional shape priors, seen in Section 2.1.3. Rüegg et al. (47) showed a similar method by ensuring predicted shapes remain discriminative of their breed by exploiting breed information classified from images. Achieving over 20% reduction in Mean Per-Vertex Error (MPVE), the average difference between the predicted 3D positions of a mesh's vertices and their ground truth positions, in their ablation study, a clear constraint is identified. Methods such as unsupervised 2D-to-3D lifting (48) help eliminate the reliance of 3D ground truth by projecting lifted 3D poses from random views back to 2D images to supervise depth estimation using adversarial 2D poses.

More recent examples include DigiDogs (49), which combines monocular 3D pose estimation with prior-aware synthetic data augmentation. The synthetic-to-real training methodology is a recurring theme across the literature, also seen by Li et al. (50) and Mu et al. (32), which have shown that carefully designed domain-adaptation strategies can provide valuable large-scale synthetic animal datasets, substantially closing the gap in performance. Trained on 8 different dogs: Labrador, Rottweiler, Shepherd, Wolf, Coyote, Poodle, Terrier, and Pug, DigiDogs aims to generalize by incorporating varied morphologies and body types. While there is variety between the included breeds, there is still susceptibility to breed-specific bias due to the limited number of samples per morphological class. The authors report an MPJPE less than 50 mm, outperforming earlier multi-view markerless baselines such as RGBD-Dogs (31) by approximately 15%, while using only monocular input. The main drawback is that poses tend to be skewed in terms of angular position, but may be remedied by other vision techniques in post processing. When interpreted in the context of fluoroscopy-validated soft-tissue artifact limits, the reported MPJPE of < 50mm places this method beyond the 10–20 mm range associated with marker-based joint center uncertainty, and within the low-fidelity category. An error of this size exceeds the joint-center localization uncertainty of clinical motion capture and is therefore unlikely to consistently preserve anatomically meaningful joint kinematics or derived gait parameters.

Outside of the obstacles discussed above, these are two major recurring issues. Firstly, occlusion is a common obstacle across skeletal based models. When objects or body parts obstruct the camera's view of certain key points, models struggle to accurately detect joint positions. To rectify this, studies have utilized multi-camera set ups to observe subjects from all angles, and reconstruct the pose with triangulation techniques (51). Secondly, a smartphone-based gait analysis system for horses showed that pose estimation models struggle with environmental conditions such as lighting conditions and terrain (52). Bright sun light can skew the detection of key points, which could be common in domestic analysis. Complex terrain, presents an issue in estimating joint positions with uneven footing or terrain not present in training datasets. This also extends to camera viewpoint dependency as many computer vision models rely on static and standardized camera positions to ensure accurate inference. Due to these limitations, real-world applications of these models often perform inadequately (38, 53).

In summary, 2D keypoint methods may support basic spatiotemporal parameter extraction under controlled views, but do not inherently provide clinically applicable kinematics or joint-center localizations in metric units. Monocular 3D keypoint methods remain constrained by occlusion and errors outside of acceptable bounds for clinical applicability.

#### 2.1.3 3D mesh reconstruction

3D mesh reconstruction methods can be split into two categories: skeletal and non-skeletal. Skeletal methods such as SMAL (45, 54), and some of its more modern implementations such as BARC (47), produce a 3D mesh that is posed via an internal kinematic skeletal structure. This results in two key outputs, 3D surface mesh of the subject and a skeleton defined by kinematic rules such as joint limits. For gait analysis, this form of output is significant as not only does it enforce important kinematic principles, it also enforces varying parameters for breeds such as bone lengths and body proportions. In contrast, pose estimation focuses on predicting a point location on the subject per-frame, which leads to base inaccuracies such as limbs bending in the wrong direction under error, generalized bone lengths leading to bias for specific breeds, and a lack of temporal consistency across frames. Non-skeletal methods focus on accurately reconstructing the surface mesh of the subject, often enforcing temporal coherence across frames which is significantly important for gait parameters such as gait cycles and cadence, alongside serving as accurate extractors for articulation through the surface mesh. While lacking a definitive biomechanical structure for holistic gait analysis, they do provide upstream stabilization for later biomechanical fitting.

##### 2.1.3.1 Skeletal

Expanding upon the models discussed toward the end of Section 1.5.1, parametric template mesh models are particularly relevant for gait analysis as they go beyond keypoints, aiming for a more kinematically coherent body representation. Parameterizing *shape, pose*, and *deformation*, these models provide a flexible 3D template that can bend and deform to represent different animal poses, breeds and bone proportions. SMAL (45, 54) is separated into 33 parts, each of which are individually controlled by shape and pose parameters, requiring parts such as joints to be optimized via inverse kinematics. This enables the encoding of canine-relevant joint limits and morphology constraints, while optimizing shape to improve subject-specific body fit, both of which are important prerequisites for stable kinematics. SMALR (55) refined this by requiring multi-view images and 2D silhouettes as input, reducing ambiguity in the 3D pose estimation. SMBLD (46) adapted the SMAL model, focusing on adding six new shape parameters to account for breed variations. SMBLD learns a detailed 3D prior from a large dog dataset (34) to ensure handling of shape and appearance diversity.

**Figure 4:**
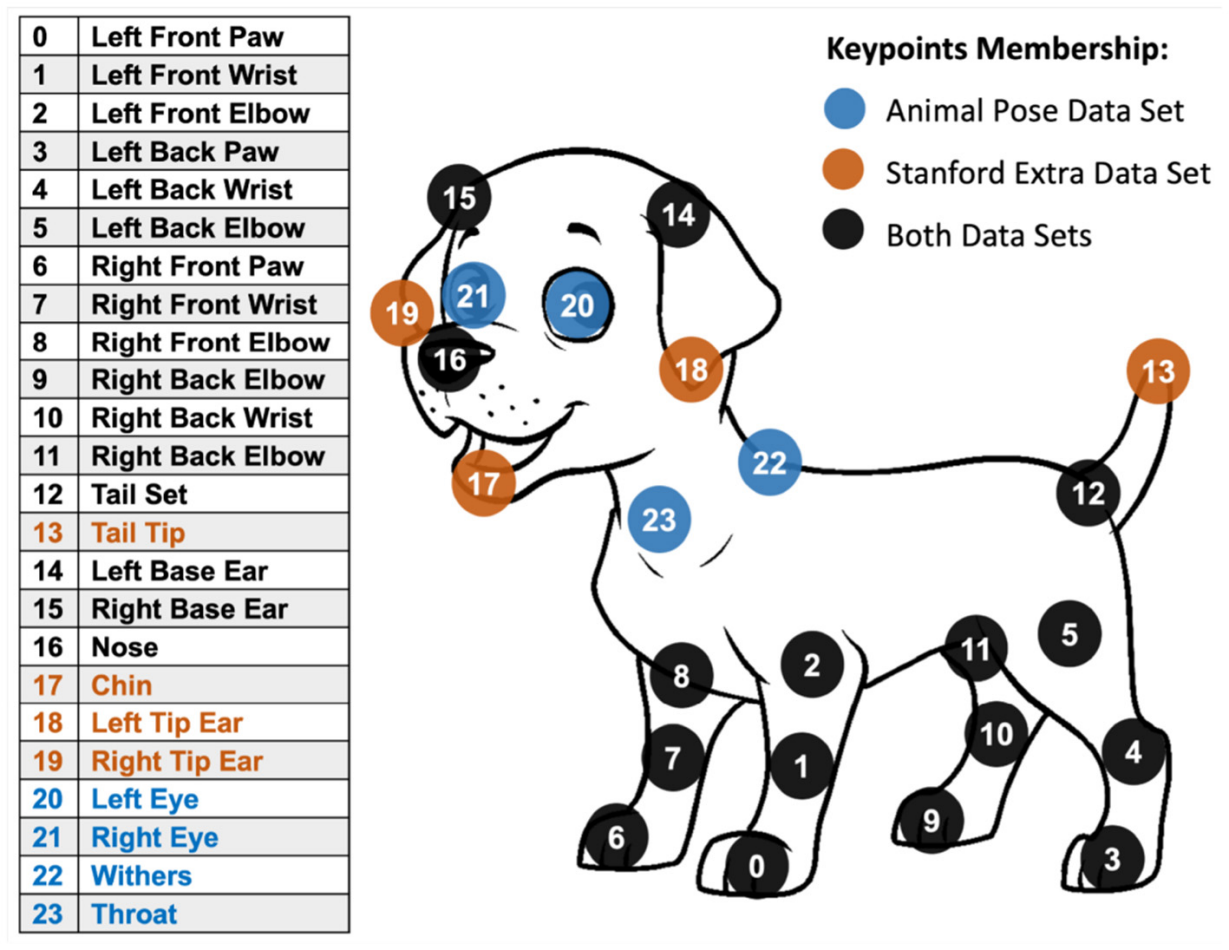
Comparison of available keypoints in popular annotated datasets for canine pose estimation from Ferres et al. (39), licensed under CC BY 4.0. The colors indicate which dataset contains the respective point.

BARC (47) focuses on 3D pose and shape regression, using breed information such as shape via breed similarity loss. BITE (56) improves upon this, exploiting ground contact points and optimizing for pose, shape, and camera translation. The resulting pose enables the derivation of 3D spatiotemporal and kinematic parameters, specifically gait cycle events such as foot strikes and lift offs can be estimated by the detection of ground contact points. As both methods are per-frame based, they are not trained/optimized with sequence-level constraints, so temporal jitter can occur without additional smoothing or video-based optimization. While the strong breed priors improve generalizability, bias may still be present due to the imbalanced training data. The authors use Stanford Dogs (as seen in Figure 4) which has over 120 breeds, but there are few samples per breed, and 3D ground-truth shapes with only showing 11 breeds, which could lead to a representation bias toward specific sizes and morphologies. For clinical gait stable joint-angle waveforms, repeatable event timing, stride-to-stride consistency are typically required, which are sensitive to this jitter. BARC primarily emphasizes 2D alignment, specifically silhouette Intersection over Union (IoU) = 75.1 and Percentage of Correct Keypoints under a given threshold (PCK@0.15) = 82.7, whereas BITE additionally 3D geometric agreement with scans: scan → mesh = 2.07 cm, mesh → scan = 3.15 cm. With missing metrics such as joint-center error or joint-angle error that we can relate to motion capture, fluoroscopy or pressure data, these methods cannot be assessed for clinical applicability from the reported metrics alone.

More recently, there have been rapid advancements through AniMer from Lyu et al. (57), which replaces the standard SMAL pipeline for a high-capacity ViT and transformer decoder alongside a family-aware supervised contrastive loss. This is reinforced by the use of their custom diffusion-generated dataset CtrlAni3D. AniMer+ (58) keeps this core, but upgrades the encoder to a ViT Mixture-of-Experts, jointly predicting SMAL (mammals) and AVES (birds). Both models report stronger accuracy and generalization than SMAL baselines. Previous examples specialize on a single toxon or use modest CNN regressors, while the AniMer unified architecture allows for shape parameters for multiple taxa to be decoded in one model. AniMer reports a Procrustes-aligned (59) MPJPE of 80.4 and Mean Per Vertex Position Error (MPVPE) of 85.7, indicating improved accuracy in 3D pose and shape reconstruction. While PA-MPJPE/PA-MPVPE are useful for comparing models, they can obscure metric joint-center errors and as such do not directly guarantee the anatomically anchored joint-center accuracy needed for gait kinematics.

Most of the previous methods outlined are temporally agnostic, whereas 4D-Animal from Zhong et al. (60) focuses on optimization from videos, using hierarchical dense cues such as silhouettes, parts, and pixel-to-vertex, to remove key point reliance and enforce temporal coherence. Consequently, 4D-Animal keeps the parametric SMAL backbone but sits between feed-forward data-prodigious approaches such as AniMer or DigiDogs, and template-fitting approaches like BARC or BITE. 4D-Animal reports mean silhouette IoU of 0.84 and a worst-case metric of IoUw5 (lowest 5% IoU across frames) of 0.71, indicating fewer failure frames under motion and occlusion. This is relevant for gait sequences as intermittent failures can corrupt stride segmentation and joint trajectory.

Taken together, BARC provides strong 2D alignment, BITE extends this with 3D geometric agreement and gait event cues via ground contacts, AniMer improves the reconstruction and generalization under Procrustes-aligned metrics, and 4D-Animal further improves robustness through video-based temporal coherence. Still, none of these studies validate gait kinematics against biomechanical gold standards such as motion capture, fluoroscopy or pressure data.

##### 2.1.3.2 Non-skeletal

Non-skeletal reconstruction methods are increasingly relevant for monocular canine gait analysis as they directly recover dense, time-consistent and metrically scaled geometry without relying on predefined skeletal templates. Such representations can serve as accurate extractors for per-frame articulation, enforce temporal coherence across frames and gait cycles, and provide metric measurements essential for spatiotemporal parameters such as stride length. Thus, they offer a complimentary foundation for biomechanical inference in scenarios where skeletal models or multi-view systems perform inadequately or are unavailable. It should be noted however, these methods lack robust evaluation for animals, in particular dogs.

Unlike pose estimation or SMAL-based mesh reconstruction methods, non-skeletal methods can provide far more accurate surface representations of subjects, particularly capturing limb articulation and body proportions. Methods such as those from Zhou et al. (61) learn to estimate 3D pose in humans from point clouds via a PointNet-style backbone (62). They report a mean Average Precision (mAP) of 80.86 under the 10 cm rule, where mAP joint prediction is considered correct if its 3D distance to the ground truth is less than 10 cm, this representing the percentage of joints satisfying this error threshold. This type of method allows for greater generalizability, removing breed-specific biases. While not based on animals, it does illustrate that accurate surface mesh alone can provide accurate articulation of limbs, alongside sufficiently strong geometric constraints to inform underlying pose estimation for biomechanical reconstruction.

Tan et al. (63) introduce a teacher network that learns per-frame neural fields that encode both the canonical shape and frame-specific deformations of an articulated object, such as a hand or animal limb. A student network is then trained via distillation to regress a compact blend-shape model, predicting per-joint skinning weights and low-dimensional shape coefficients directly from a single input observation. The separation of geometry and time-varying deformation imposes implicit temporal consistency, as all frames are constrained to deform from a common template. In parallel, Song et al. (64) address deformable scene reconstruction, and Yang et al. (65, 66) focus on learning the reconstruction of animatable 3D neural models and categories from casual videos. These four approaches are unified in Lab4D (67), which provides an open-source framework for reconstructing fully articulated, time-varying 3D shapes from a single RGB video. These methods are less generalizable than pointcloud-based methods due to the prediction of skinning weights. Similar to SMAL-based methods, this form of parameterization is limited to the training dataset, and in this case its generalized to 3D shapes rather than dogs and their varying breeds. The shared canonical geometry and deformation parametrization across frames enforce temporal coherence in the reconstructed geometry and motion. However, rather than being constrained by biomechanical principles, these models are optimized to explain visual appearance and temporal continuity of the observed shape and motion. These temporally consistent representations are critical for a gait analysis, particularly when considering gait cycles, cadence and other spatiotemporal parameters.

Keetha et al. (68) have shown significant results in metric scene reconstruction with minimal data input. Inputs have been reduced to allow for any combination of monocular/stereo RGB images, calibrations, camera poses, and depth, while still outputting predicted geometry outputs (point-clouds), camera intrinsics and extrinsics in metric scale using a unified vision-transformer backbone, thereby enforcing a coherent geometric reference frame across views and time. This metric consistency is particularly valuable in monocular capture scenarios where absolute scale is ambiguous, providing a necessary foundation for spatiotemporal gait measurements such as stride length and walking speed in metric scale. Similar to Zhou et al. (61), MapAnything is breed agnostic due to the point cloud being built from depth maps, allowing for generalizability across morphologies.

In summary, non-skeletal reconstruction methods provide metrically accurate, temporally coherent surface representations that can give strong geometric constraints for underlying skeletons. Although not specifically biomechanical, these give metric scale to improve accuracy of metric spatiotemporal measurements, surface continuity that provide accurate pose representations and outer bounds for skeletal fitting, and temporal consistency for gait analysis, making them a valuable upstream component for monocular canine gait analysis.

### 2.2 Gait analysis

To perform accurate gait analysis on a subject, one requires expert subjective visual assessment, alongside objective kinematic and spatiotemporal analysis. As mentioned previously, these are typically performed in clinical settings by way of the methods in Section 1.4. All of these methods produce various parameters, such as those seen in Table 2. Which parameters are required varies widely depending on the desired analysis i.e., lameness detection, diagnostics, rehabilitation, etc. Most research offer a general approach, often relying on abnormality detection (69). A summary of key publications in the literature can be seen in Table 6.

**Table 6:** Overview of gait analysis methods which utilize computer vision and machine learning.

References	Species	Category	2D/3D	Clinically verified
Liu et al. (79)	Multiple	Limp detection	2D	Partial
Palez et al. (81)	Dogs	Multiclass & diagnosis classification	3D	Yes
Gill et al. (75)	Dogs	Parameter extraction	2D	Partial
Gong et al. (53)	Quadruped	Parameter extraction	2D	No
Feuser et al. (52)	Equine	Gait pattern classification	3D	Yes
Wong et al. (71)	Rodent	Manual	3D	N/A
Silva et al. (77)	Sheep	Parameter extraction	3D	No
Verlekar et al. (112)	Human	Abnormal gait detection	2D	No
Jameel et al. (113)	Human	Biometric identity gait recognition	3D	N/A
Nieto-Hidalgo et al. (83)	Human	Abnormal gait detection	2D	No
Sipari et al. (114)	Human	Parameter extraction	3D	Yes
Mehrizi et al. (89)	Human	Abnormal gait detection	3D	No
Greve et al. (115)	Human	Parameter extraction	3D	Yes
Prakash et al. (73)	Human	Parameter extraction	2D	No
Kondragunta et al. (74)	Human	Parameter extraction	2D	No
Ng et al. (85)	Human	Abnormal gait detection	2D	Yes
Guo et al. (69)	Human	Abnormal gait detection	3D	No
Sanchez-DelaCruz et al. (88)	Human	Gait pattern classification	3D	No
Potluri et al. (80)	Human	Abnormal gait detection	3D	No

Due to the scarcity of research specific to canines, and even quadrupeds, various species were included as part of this table, including: quadrupeds, equines, rodents, sheep and humans. The aim is to provide an overview of the state-of-the-art for vision-based gait analysis methods, showing their applicability to vision-based automatic canine gait analysis.

For vision-based systems to be considered suitable for clinical gait analysis, a set of minimum validation requirements must be satisfied. From a kinematic perspective, joint trajectories and derived spatiotemporal parameters should show agreement with gold-standard systems such as marker-based motion capture, treadmill and pressure walkways, or fluoroscopy-validated multibody models, with errors remaining within the intrinsic uncertainty bounds imposed by soft-tissue artifacts. At a clinical level, extracted gait metrics should show correlation with established outcome measures and diagnostic reference, including CMIs (Section 1.4.6). Lastly, validation should be demonstrated across a representative range of breed morphologies to ensure robustness and generalizability beyond laboratory-based scenarios. In the human field, key metrics that are typically tracked in the literature are Mean Per-Joint Position Error (MPJPE) or Joint Localization Error (JLE), Mean Per-Joint Angle Error (MPJAE), Standard Error of Measurement (SEM), Minimal Detectable Change (MDC), and intraclass correlation coefficient (ICC).

Data collection is a common issue for these types of methods. While multiple public datasets such as AP10K (70) can be useful, they lack “trainable” data from a gait analysis perspective. Furthermore, they also lack the expert-validation typically seen in clinical contexts. The use of traditional methods, as outlined in Section 1.4, can serve as solid ground truth data for these gait analysis systems, shown by Wong et al. (71). Feuser et al. (52) took this one step further by clinically validating their data using an expert lameness examination, based on the AAEP scale (72). Gong et al. (53) captured their own data by way of tripod-fixed shooting gear with rigid capture conditions such as camera height and a fixed motion direction angle of –33° to 33°. While these conditions aided in the research, they lack the generalizability for domestic scenarios. Alongside this, they only focused on set parameters: gait frequency, gait sequence recognition, gait duty cycle and gait trajectory. While each of these are valuable, they are not clinically validated and do not provide a holistic perspective on the subjects gait. Breed-related morphological variability also significantly influences gait analysis. For instance, chondrodystrophic breeds, characterized by shortened limbs and elongated trunks, exhibit kinematic normals which may vary when compared to dolichocephalic or other morphologies. This may produce a scaling bias unless accounted for with breed-agnostic or generalizable data.

#### 2.2.1 Parameter extraction

Gait parameters are fundamental to gait analysis as they provide quantifiable measures of an subject's movement pattern. These parameters, as seen in Table 4, offer precise insights into the mechanics of movement, enabling clinicians and researchers to develop targeted interventions, monitor progress, and ultimately improve patient outcomes. These parameters can be extracted through calculation, time-series analysis or predicted by a machine learning system. Prakash et al. (73) focused on the use of silhouette images as an input for skeletonization and gait parameter extraction. The parameters extracted include: Crotch Height, Foot Length, Step Length, Stride Length, Cadence, Cycle Time, and Cycle Distance. A clear obstacle seen in this study as the focus is on the sagittal view, vastly reducing the amount of parameters extractable and therefore not providing a holistic view into the subject's gait. Despite this, the parameters extracted showed good accuracy and the methods used, if integrated into modern vision-based solutions, could serve as an effective way of viewing a subject's gait characteristics. Kondragunta et al. (74) used a more modern method of parameter estimation by using 3D pose key points as inputs for calculating parameters such as step length, stride length, swing time, stance time, stride time, and cadence in elderly humans. Heel joints from selected frames are analyzed to calculate their horizontal displacement using Euclidean distance. A uniform kernel convolution smooths the gait trajectory, helping identify the gait's maximum and minimum points for parameter estimation. While this study does not focus on techniques of analysis, the ability to accurately extract the parameters is invaluable for later analysis of the subject's gait.

Focusing on quadrupeds, Gong et al. (53) created separate models for gait frequency extraction, gait sequence recognition, duty cycle extraction and trajectory extraction. Their pose estimation used keypoint localization via object keypoint similarity rather than typical MPJPE, following the COCO/AP10K protocol of related detection metrics (AP, mAP, and AR). This results in a dimensionless similarity score rather than a physical joint-center error expressed in millimeters or degrees, therefore making biomechanical anatomical fidelity difficult to evaluate. Models for gait parameters use relative error *E*_*rel*_, comparing extracted values with manually annotated ground truth, reporting a maximum gait frequency error of 2.46% and maximum error of duty cycle at 4.33%. While these results initially look promising, they lack clinical validation, primarily because the reference ground truth consists solely of manual frame-by-frame video annotation, without comparison to fluoroscopy or marker-based motion capture. More importantly, there is no gait ground truth such as force plates, nor correlation with clinical outcome measures or CMIs, meaning it cannot be assessed for clinical validity from the reported metrics alone. Further, the reliance on 2D plane estimation and an absence of depth data preclude accurate reconstruction of joint kinematics and inter-limb symmetry, resulting in the loss of potentially clinically relevant information. Consequently, it does not validate whether the inferred joint locations would fall within the 10–20 mm anatomical error bounds required for biomechanically interpretable kinematics, nor whether extracted duty factor or frequency would correspond to force-plate-derived symmetry indices or temporal parameters.

Gill et al. (75) utilized the popular DeepLabCut model (38) with a single lateral-view camera to extract 2D gait parameters from the sagittal view. Similar to Gong et al. (53), they use a Median Absolute Euclidean Distance (MedAED) per joint landmark. Although pixel errors are low at 0.55% and size-normalized. they are not converted to millimeters and thus cannot be directly assessed against the 10–20 mm threshold previously defined. They do note, however, that a RMSE inflation for the hip and stifle in deerhounds demonstrates loss of anatomical fidelity in out-of-distribution morphologies. Identifying the gait cycles, they extracted key kinematic and spatiotemporal parameters, comparing both duty factor and stifle range of movement for Labradors with published data from the literature. Results proved promising, with metrics such as walking velocity (0.65 ± 0.08 m/s) being more consistent than those reported in pressure-walkway studies (76). Although the median values aligned with the literature, their results showed a broader distribution of measurements, reflecting the variability produced by markerless tracking and morphological diversity. Furthermore, validation is limited to comparison with previously published kinematic means, without reference to force-plate kinetics, fluoroscopy, or clinical outcome measures (e.g., lameness scores or CMIs), preventing assessment of diagnostic sensitivity or validity. With respect to bias across breeds and shapes, their utilization of pose estimation trained on AP10K introduces a shared skeleton across varying animals due to the dataset being trained on multiple species. As mentioned by the author, poor performance due to environment, pose and hair characteristics show a pathway for bias of breeds with darker or heavy coats.

Silva et al. (77) utilized a marker-based motion capture system with infrared cameras to track retro reflective markers placed on sheep's key anatomical landmarks on both hindlimbs. The data is collected while the sheep walk along a designated track, ensuring natural movement patterns. Considering sheep as a quadruped biomechanical analog, parameters were calculated based on the kinematic markers which could be emulated via 3D pose estimation. This approach ensured precise and directly observable measurements of gait characteristics from the subject. This configuration yielded full 3D joint kinematics in flexion/extension, abduction/adduction and internal/external rotation, with joint angle ranges such as knee flexion reaching 80.6°± 11.8° at mid-swing and ankle dorsiflexion 80.7°± 11.5°, showing a high-fidelity reference for spatiotemporal and angular gait parameters. Parameters were calculated directly from the reconstructed kinematic marker trajectories, constituting gold-standard joint center and segment orientation measurements against which vision-based 3D pose estimation systems could, in principle, be quantitatively validated. The authors report stance and swing phase variability (e.g., hindlimb stance 0.373 ± 0.047s left, 0.378 ± 0.091s right), demonstrating that even under controlled laboratory conditions inter-cycle variability on the order of 10–15% is present in temporal parameters. This laboratory environment, while very useful for gold standard tracking, introduces the environment as a secondary variable that can significantly alter joint kinematics and ground-reaction forces in quadrupeds, as seen from Feuser et al. (52) and Jarchi et al. (78). While these same environmental and behavioral influences may affect vision-based systems, it would allow for longer term data acquisition in familiar domestic environments, reducing the influence it may have on biomechanics. Together, these motion-capture results illustrate the level of spatial, temporal, and angular precision that vision-based systems must achieve, and validate against kinetic and clinical ground truth before they can be considered suitable for clinical gait assessment.

#### 2.2.2 Abnormal gait detection

One of the most logical approaches to gait analysis in a subject is the differentiation of normal and abnormal gait as a first step toward clinical screening and diagnostic support. The literature presents a number of obstacles in establishing whether such differentiation is clinically reliable and biomechanically meaningful. Firstly, the classification and analysis of gait must be designed based on a desired outcome and as such requires a dataset specific to this need, such as sound gait alongside disease or condition-specific abnormal gaits that are confirmed by clinical examination and, where available, objective reference standards. Naive methods such as those seen by (79) take a “Healthy vs. Limping” approach by creating simulated limp states within their datasets and training a YOLOv5 deep CNN to predict the binary classifications. These methods yielded 97% accuracy for normal walking states and 70% accuracy for limping states under this simulated labeling scheme. While their dataset assessed a variety of species, this simple approach shows preliminary promise, without the use of any pose data. This variety of species also means that per sample occurrence can induce bias for specific morphologies. The key pitfalls in this method are a lack of skeletal gait data, and more importantly a simulated dataset that has no kinematic basis and therefore cannot be directly validated against clinically interpretable joint kinematics, spatiotemporal parameters, or veterinarian-assigned lameness grades.

Inertial sensors, as seen in Potluri et al. (80) provide direct access to segment-level kinematics and temporal gait events, specifically in humans, but require condition-specific abnormal gait data that is labeled according to clinically defined pathologies. Palez et al. (81) partially address these limitations by utilizing IMUs for 3D kinematic data, and a deep learning classifier to predict whether a dog's gait is healthy, orthopedic, neurological, or (binary) healthy vs. non-healthy. The multi-class classification (healthy vs. orthopedic vs. neurological) achieved up to 96% accuracy on the random-split baseline, while differentiating orthopedic and neurological lameness had lower performance due to an overlap of clinical signs and shared compensatory kinematic patterns. Similar to previous literature, the lack of a wide dataset produces a class imbalance as well as difficulty generalizing across morphologies and pathologies, highlighting the need for validation across breed groups and disease severities before such classifiers can be considered clinically robust.

Depending on which abnormalities are being investigated, some can only be viewed from a frontal plane while others from a sagittal plane (82, 83), implying that clinically reliable abnormality detection requires multi-view or view-specific acquisition. In the case of Nieto-Hidalgo et al. (83), promising results were produced by the use of smartphone data capture and cloud-based inference for spatiotemporal feature extraction, but with clear limitations in both abnormal gait detection as a method of holistic analysis and the lack of specific datasets for different morphologies and varied pathological gait patterns, thereby preventing systematic validation across breed conformations and disease-specific kinematics. For clinical applications, classifiers must demonstrate not only high overall accuracy but high sensitivity to asymmetric and low-grade lameness, and stability across repeated trials and environments.

Guo et al. (84) focused on 3D pose estimation with the extraction of gait parameters for abnormal gait detection using clinically interpretable kinematic features rather than silhouette-level descriptors. Rather than typical binary classification performed by skeletal analysis, they focused on recognition tasks for six gait patterns (normal, in-toeing, out-toeing, drop foot, supination, and pronation) corresponding to clinically distinct movement deviations. The use of depth sensor cameras allowed for an accurate 3D estimation of human pose alongside the 6D Vicon (8) motion capture system which was used for ground truth data, thereby enabling direct validation against a gold-standard kinematic reference. While results under 62% accuracy were achieved, this is largely due to a lack of normalization between body shapes and proportions, implying that morphological variability directly degrades abnormal gait classification performance, despite the use of gold standard motion capture. This presents another obstacle which would be even more challenging in dogs due to wide breed size and variation, where bone length ratios, joint orientation, and trunk proportions vary far more than humans. Breed classification would be necessary to normalize gait patterns across sub-species (47), and should as such be considered a prerequisite for clinically valid abnormal gait detection.

Ng et al. (85) approached this in two key steps: the extraction of gait parameters and the comparison of the gait data with clinical mobility assessments like the Performance-Oriented Mobility Assessment (POMA) (86), showing deviations from the normal gait relative to established clinical scoring criteria. Due to the clinical nature of POMA, this also offers a form of clinical validation that the gait analysis method works by directly linking quantitative kinematic deviations to clinical impairment scores. Although this study focused on human subjects, the same validation mechanism can also be applied to canines using equivalent veterinary outcome measure such as LOAD (87) or other CMIs (see Table 1). A similar traditional method can be seen in dogs (6) through spatiotemporal gait analysis with pressure sensor walkways. While relying on external equipment, many of the same kinematic parameters are used which could provide a viable point of comparison similar to POMA for correlating vision-based gait metrics with clinically accepted metrics such as symmetry indices, stance time distributions, and limb loading patterns.

#### 2.2.3 Gait pattern classification

Gait pattern classification functions in a similar manner to abnormal gait detection, whereby a focus is put on certain gait patterns that are indicative of specific conditions or diseases, and therefore has direct relevance for clinical applications such as differential diagnoses and longitudinal monitoring. There has been extensive research in the use of machine learning for the classification of patterns based on a specific set of gait parameters, but these parameters are mostly confined to clinical environments and the use of external equipment such as inertial sensors or motion-capture and force-plate systems which provide kinematically and kinetically validated ground truth. The challenge lies in the extraction of gait parameters via vision-based techniques without the use of external equipment, sensors or laboratory environments, while still maintaining sufficient accuracy and repeatability for clinical applications.

Sanchez-DelaCruz et al. (88) assembled 23 classifiers combined with a deep learning algorithm to classify gait bio-markers for pathologies associated with diabetic neuropathy, using biomechanical data from accelerometers as established clinical indicators of nerve and muscle dysfunction. Unlike much of the previous literature, the researchers managed to achieve a clinically validated accuracy of over 85% against physician-labeled diagnostic categories. Unfortunately, this was achieved by the use of inertial sensors rather than vision-based methods, and therefore does not establish whether comparable performance can be achieved when gait biomarkers must be inferred from video-based joint kinematics. Overcoming this obstacle, Feuser et al. (52) demonstrated forelimb lameness by observing indicative and iterative head nods compared with sound horses using pose-estimated trajectories of clinically validated anatomical landmarks and reference grading according to the AAEP lameness scale (72). The analysis showed stifle kinematics provided more reliable information, whereas tuber coxae displacement gave greater variability.

In humans, Mehrizi et al. (89) used abnormal gait detection in humans to first decide whether a gait was considered healthy or not, and then a deep neural network and window slicing of the time-series to classify the gait pattern present into four pre-defined categories: Healthy, Parkinson's disease, Post Stroke, and Orthopedic disorders. This was accomplished using 3D joint trajectories estimated from video and validated against a marker-based motion capture system, thus demonstrating a pipeline in which the pose accuracy must be accurate enough for clinical analysis before it can be use to reliably classify disease-related gait patterns. The pose accuracy was quantified by average Euclidean distance between estimated 3D joints and marker-based motion capture joints (MPJPE), reporting an average of 36.12 ± 17.41*mm*. While this level of error exceeds the 10–20mm accepted bounds, it was still sufficient to support robust disease-level gait pattern classification, reporting misclassification as very low 1/25 healthy misclassified and 1/70 patients misclassified as healthy.

In summary, the initial extraction of gait parameters, followed by abnormal gait detection and pattern classification, combined with 3D skeletal pose estimation methods, provides a comprehensive roadmap for automatic gait health evaluation in canines. It is not enough to look at parts-based analysis, we must capture and analyse gait data across spatial, temporal and inter-limb coordination for a subject. As we know the means are available to create parts-based views into a subject's locomotive function, the process of combining this information and then producing an actionable health score evaluation, or clinically interpretable indicators, is imperative for use in clinical and domestic settings. This highlights a clear avenue for future work in integrating multi-level kinematic descriptors into unified clinical inference frameworks. Aside from this, a clear need for interdisciplinary collaboration is seen between veterinary science, biomechanics and computer vision. Ensuring clinically/expertly verified methods and data is essential for use in the medical sector and for establishing minimum validation standards, ground-truth reference protocols, and outcome-linked performance metrics. Working with veterinary and other medical collaborators also provides more in-depth context surrounding specific parameters associated with gait analysis.

## 3 Results

The comparative examination of traditional and computer vision-driven techniques presented in Sections 1.4, 2 reveals that current vision-based pipelines reproduce spatiotemporal trends but do not yet achieve the biomechanical accuracy or clinical validation of marker-based systems. Rather than viewing these as isolated methods, we draw a clearer picture when considering how articulated pose and shape reconstruction, parameter extraction, and gait interpretation interact to form an end-to-end pipeline.

### 3.1 Emerging requirements

Across the reviewed literature, three technical requirements consistently surface when evaluated against the biomechanical accuracy limits and clinical validation criteria discussed in Sections 2.1, 2.2:

- A robust monocular 3D articulated shape and pose reconstruction pipeline, capable of handling varying canine morphologies, occlusion and real world environments, while achieving joint localization and angular accuracy within the soft-tissue artifact bounds required for biomechanically interpretable kinematics (10–20 mm).
- A clinically meaningful set of gait parameters, derivable from 3D pose sequences, aligned with clinical metrology instruments and gait assessment standards, ensuring they agree with gold-standard measurement from pressure walkways, motion capture or fluoroscopy.
- A move from parts-based to holistic gait analysis, where discrete parameters are integrated to generate an interpretable and clinically-relevant output, enabling correlation with clinical outcomes and CMIs.

These requirements directly reflect the limitations and research gaps exhibited across published literature, and define the minimum methodological and validation criteria that vision-based gait analysis systems must satisfy for clinical applications.

### 3.2 Trends observed in 2D and 3D pose estimation

The literature shows a clear trend: while 2D pose estimation continues to be an accessible and useful entry point, it fails to preserve critical kinematic detail seen in 3D. For example, parameters involving the spatial orientation of joints cannot be reliably extracted when depth information is missing. This is consistent with Fischer (21), who emphasizes that 2D projections distort limb orientation and can induce false symmetry or stride patterns. In contrast, 3D pipelines such as DigiDogs (49) demonstrate an imperfect monocular reconstruction which can still produce joint trajectories and orientations that permit estimation of clinically relevant 3D kinematic parameters over state-of-the-art 2D approaches. However, angular drift, breed-related shape bias, and generalization failures lead to joint localization and orientation errors that exceed the 10–20 mm biomechanical fidelity range required for clinically interpretable kinematics. The consistent reliance on synthetic data also introduces domain gaps which affect downstream gait interpretation.

Taken together, these trends indicate that future progress depends not only on improving pose accuracy but on creating canine-specific, anatomically grounded 3D priors and more diverse real-world training data, aiming to ensure accuracy remain within the acceptable uncertainty bounds imposed by soft-tissue artifacts.

### 3.3 Extractable gait parameters

By cross-referencing the gait parameters shown in Table 4 with published pose estimation outputs, a clear delineation emerges:

- 2D systems reliably extract only spatiotemporal parameters and show limited robustness when estimating parameters such as rotation, pelvic motion or elevation.
- 3D systems enable a broader set of three-dimensional kinematic parameters (e.g., joint angles, pelvic motion, and inter-limb coordination) that are essential for clinically aligned gait interpretation and analysis.

Although 2D pose estimation can provide the basic spatiotemporal parameters, its accuracy rapidly deteriorates under real-world conditions such as occlusion, variable lighting and viewpoint changes, as highlighted across the literature (38, 52). These limitations lead to joint localization errors that frequently exceed the anatomical uncertainty bounds previously defined, particularly when limbs are partially obscured or when camera placement deviates from standardized setups. As Fischer (21) emphasizes, 2D projections distort depth and joint orientations, making them unsuitable for capturing the full 3D motion required for clinically meaningful gait interpretation and analysis. Together, the evidence clearly suggests that reliable canine gait analysis depends on true 3D skeletal reconstruction or mesh-based representation that can accurately preserve spatial and anatomical relationships, while remaining within acceptable soft-tissue artifact bounds.

### 3.4 Toward integrated gait analysis

Progressing from vision-based parameter extraction to clinical interpretation in canines represents an area in the current literature that has been explored to a limited extent, particularly with respect to outcome-linked clinical validation. Most studies stop at reporting parameter accuracy or mean joint position error, without demonstrating agreement with gold-standard kinetic measures, fluoroscopy-validated kinematics, or correlation with Clinical Metrology Instruments (CMIs) and veterinarian-assigned lameness grades. A small number of studies provide promising precedents, such as condition-specific classification (52, 81, 88), or lameness detection (79), yet these typically focus on single parameters, controlled environments, or specific camera angles such as lateral views, limiting their ability to capture inter-limb coordination or compensatory movements.

Throughout the literature, it becomes clear that gait analysis in canines is inherently multifaceted. Parameters interact non-linearly, pathologies manifest variably across breeds, and environmental factors (lighting, terrain) influence gait profiles. This level of complexity strongly suggests the need for ensemble or multi-module machine learning frameworks, where different aspects of the gait cycle are analyzed independently and then fused to produce a holistic assessment that supports clinically interpretable, repeatable, and morphology-aware inference across gait cycles and environments.

### 3.5 Comparative analysis

Pose estimation and reconstruction methods outlined in Section 2.1 provide a view of the current technical landscape as it pertains to extracting data usable for gait analysis. Throughout, we identify outputs from given models, their applicability for extracting gait parameters, and evaluate their applicability in clinical gait analysis. Table 7 presents these methods in a comparative format, specifically comparing accuracy metrics, validation against gold standard methods, breed robustness and extracted parameters. These studies were chosen as representative methods due to their accuracy and applicability to clinical gait analysis. As it pertains to accuracy metrics, there is a clear divide between studies in how they evaluate the performance of their respective models. Most utilize within-dataset error measurements such as MPJPE, PA-MPJPE, and AP, which often cannot be interpreted clinically because the ground truth is not biomechanically validated against gold standard methods such as motion capture or fluoroscopy for joint-center localization. They also do not all follow the same accuracy metric, with skeleton-focused studies commonly reporting MPJPE (or Procrustes-aligned variants), whereas mesh/shape reconstruction approaches more frequently report overlap or surface-based measures (e.g., IoU), reflecting different task objectives and error definitions.

**Table 7:** Comparative analysis of key methods for vision-based pose estimation and reconstruction, with an emphasis on canines.

Method	Accuracy metric	Gold standard validation	Breed robust	Extracted parameters
Lyu et al. (57, 58)	PA-MPJPE: 80.4, MPVPE: 85.7	No	Yes	Shape & skeleton
Xu et al. (36)	AP: 79.4 (COCO), 76.8 (AP10K)	No	No	2D keypoints
Shooter et al. (49)	MPJPE < 50 mm	No	No	3D keypoints
Ruegg et al. (56)	Scan → Mesh: 2.07-3.15 cm	Yes	Yes	Shape, skeleton & ground contacts
Ruegg et al. (47)	IoU 75.1%, PCK 82.7%	No	Yes	Shape & skeleton
Biggs et al. (46)	IoU: 67.5	No	Yes	Shape & skeleton

Due to the scarcity of canine-specific research, this table includes state-of-the-art methods applied to other species also. Studies are compared based on accuracy metrics, validation against gold standard measurements (e.g., motion capture, 3D scanning), breed robustness, and extracted parameters.

Several methods report robustness across breeds and varying morphologies, particularly BARC (47) and BITE (56) which incorporate breed priors as part of their shape and pose optimization framework. Methods that incorporate SMAL templates, such as these, have the benefit of instance-specific reconstruction, rather than a generic shape to represent all animals. BARC has the added feature of automatically detecting ground contacts, which provides an initial set of gait parameters to track gait cycles via foot strikes and lift-offs, enabling the extraction of stance and swing timing.

Table 8 synthesizes vision-based gait analysis studies with applicability to canines, comparing accuracy metrics, clinical validation, robustness to breeds and extracted parameters. These studies were chose as representative methods to show the variety of extracted parameters, alongside their clinical validation and accuracy. Only a subset of these studies report full clinical validation, Feuser et al. (52) validate against the AAEP lameness scale for horses (72), while Palez et al. (81) performed rigorous assessments on canine patients with a subset receiving MRI scans for neurological disorders. Palez et al. also utilized a pressure-sensing walkway during data collection but this was not the primary validation comparator, instead opting for a clinical diagnostic label defined from assessments. Relatively few studies emphasize continuous kinematics such as displacement and range of motion (ROM), both Palez et al. and Liu et al. (79) focus on binary or multi-class classification rather than parametrized gait assessment. Classification-based methods report strong performance but may offer limited clinical interpretability beyond categories, whereas kinematic/spatiotemporal methods provide more clinically meaningful parameters but often face strong constraints from measurement validity. Lastly, breed robustness is generally not reported, leading to a lack of generalization across varying morphologies.

**Table 8:** Comparative analysis of key vision-based gait analysis studies, with an emphasis on canines via images or video.

Method	Accuracy metric	Clinical validation	Breed robust	Extracted parameters
Gill et al. (75)	MedAED (SD): 0.14–0.24	Partial	No	Duty factor, stifle ROM
Palez et al. (81)	Acc: 96% (multiclass), 82% (binary)	Yes	Partial	Gait classification
Liu et al. (79)	Precision: >0.95, Confidence: >0.96	Partial	No	Binary gait classification
Gong et al. (53)	*E*_*rel*_: 2.46% Gait Freq., 2.41% Duty Cycle	No	No	Gait frequency & Duty cycle
Feuser et al. (52)	SE: 100% (forelimb), 88.9% (hindlimb-stifle); SP: 100%, 87.5%	Yes	No (Horses)	Head trajectory, stifle displacement

Due to the scarcity of canine-specific research, this table includes state-of-the-art methods applied to other species also. Studies are compared based on accuracy metrics, clinical validation status, breed robustness, and extracted gait parameters.

## 4 Discussion

The reviewed literature shows a rapidly evolving intersection between computer vision, biomechanics and veterinary science, where methodological advances in pose estimation, mesh reconstruction, and gait analysis are starting to enable objective assessment of canine locomotion. Translating these advances from controlled laboratory settings with gold-standard capture to uncontrolled video capture in domestic environments offers unprecedented opportunities for longitudinal and large-scale analysis. Considering this, we also see key limitations in current methods with respect to anatomical fidelity, validation, and clinical applicability. This section synthesizes the key findings from the reviewed literatures, implications for automated canine gait analysis, ethical considerations, future research directions, and the broader significance of computer vision in canine and animal research.

### 4.1 Main findings from the literature

#### 4.1.1 3D pose is essential for clinically meaningful gait interpretation

While 2D pose estimation continues to be widely used, the literature consistently shows that 2D projections cannot extract the kinematic detail necessary for clinical assessment. Additionally, 2D projections distort depth and joint orientations, resulting in missing compensatory movements and key joint mechanics such as the forelimb pivot being located at the upper level of the scapula, not level with the hip joint, as discussed by Fischer et al. (21). Monocular 3D reconstruction methods, while imperfect, may provide substantially more clinically relevant motion information.

#### 4.1.2 Current datasets limit real-world applicability

Most of the studies review rely on small, homogeneous datasets, often collected under controlled conditions and/or inconsistent breed variation. The larger datasets are typically synthetic in nature, relying on domain adaption to accurately apply to “in the wild” scenarios. This creates a significant domain gap when models are applied to domestic dogs in home or outdoor settings. Breed diversity remains particularly underrepresetned, with exception to methods such as BITE (56) and BARC (47).

#### 4.1.3 Methods are not validated against a clinical applicability framework

Literature based on clinical movement analysis use a number of key performance metrics to establish applicability to clinical applications. Vision-based methods are currently not compared to these metrics, largely due to the difference in disciplines. These metrics, once gathered into an acceptable framework, provide a clearer inter-disciplinary relationship which could be used to validated acceptable error ranges, their relationship with soft-tissue artifact limits, and their implications for kinematic and spatiotemporal parameter extraction.

As previously stated in the context of fluoroscopy-validated soft-tissue artifact limits, ~10–20 mm joint center error from markers is acceptable as it still retains interpretable kinematics. Errors exceeding this approach the magnitude of skin-bone motion itself and indicate a loss of anatomical fidelity. In human studies, acceptable joint-angle error bands are 2–5° (90). As veterinary consensus thresholds are not yet standardized, these currently provide the only available quantitative guideline. Using Minimum Detectable Change (MDC) provides important context as to why these error bounds matter. If a markerless approach produces errors in the same range as the MDC or standard measurement error, pathology-related changes may be indistinguishable from noise, therefore limiting the systems utility for pathology detection or longitudinal monitoring. The lack of validation represents the most substantial gap between academic prototypes and clinically deployable systems.

Beyond pose accuracy and biomechanical fidelity, ensuring that measurements are repeatable under the same conditions is paramount for clinical applicability. Throughout the literature, specifically Koo et al. (91) and Aghapour et al. (92), this is most commonly quantified as the Intraclass Correlation Coefficient (ICC), which evaluates the proportion of total variance due to biological variation, rather than just measurement noise. According to established guidelines, ICC values below 0.5 indicate poor reliability, 0.5–0.75 moderate, 0.75–0.9 good, and above 0.9 excellent reliability. While vision-based literature focuses on accuracy metrics such as MPJPE, clinical viability must be evaluated through extended means such as ICC with confidence intervals and inter-rater reliability.

### 4.2 Implications for automated canine gait analysis

Together, these findings collectively highlight the primary barrier to progress is not solely improved pose accuracy, but the requirement for frameworks that combine pose, gait parameters and clinical applicability. Several implications are suggested by an analysis of the literature. Firstly, biomechanical interpretability and clinical applicability should be treated as a primary design constraint, rather than a downstream add-on. Common computer vision-based evaluations such as MPJPE, when reported without absolute metric reference, do not provide direct comparison to ground truth or gold-standard measurements such as fluoroscopy or motion capture. Establishing a standard threshold for joint angle error, as in human methods (90), and incorporating validation measures used in veterinary literature, such as ICC and MDC, would therefore allow for a more clinically meaningful assessment of pose estimation and gait analysis systems.

Secondly, model design should either be breed-agnostic or breed-specific, with accurate dataset curation to avoid systemic bias. Current methods such as BITE (56) and BARC (47) have incorporated this as a prior for SMAL-based mesh and skeleton reconstruction, reporting more generalizable applicability. This is essential as varying morphologies lead to an adaptability problem for most models, losing accuracy due to varying coat lengths, body proportions and bone lengths.

Thirdly, a lack of ground-truth datasets makes it difficult to compare the accuracies of different methods or their overall biomechanical precision. The literature has suggested that measurements within ~10–20 mm of joint centers measured with fluoroscopy are sufficient for retaining kinematic accuracy. As such, an accessible motion capture or fluoroscopy dataset would provide comparative reference to known bounds. However, due to high costs and logistical difficulty this may not be feasible, thus utilizing synthetic datasets and augmentation may be a valuable alternative, but insufficient without accuracy for varying coats, lighting, environmental diversity, and natural motion. If synthetic datasets could be further refined to include these, as well as represent varying morphologies, body conditions and accurate biomechanical simulations, then their value would greatly increase. It should be noted however, due to domain gap issues discussed in the literature, moving from synthetic training to real-world inference often introduces systemic bias. As such, more accurate domain adaptation would also be required.

Lastly, models should integrate temporality as context, due to many abnormalities and pathologies manifesting across gait cycles rather than single frames. The literature has presented many varying methods to retrieve biomechanical representations, but few incorporate the temporal consistency and smoothness to reflect actual gait. Looking to methods such as Lab4D (67) may provide key insights into how we may incorporate this temporal cohesiveness into current state-of-the-art methods.

### 4.3 Ethical considerations

Capturing images or videos of canines in domestic settings for the purpose of gait analysis presents a number of ethical considerations. Videos may inadvertently contain personal identifiable information such as human faces, residential addresses, identity documents, and vehicle registration plates. Before considering specific technical mitigation strategies, the collection and processing of user-uploaded canine videos must be framed within data protection and research ethics frameworks. Principles such as data minimization and purpose limitation would require that only information relevant to gait analysis is retained, while transparency and informed consent drive the usage, clearly communicating how videos are processed, stored, and potentially reused. Key regulatory frameworks such as General Data Protection Regulation (GDPR) and analogous health data protection frameworks like the Health Insurance Portability & Accountability Act (HIPAA), alongside ethical guidelines from bodies such as the American Veterinary Medical Association (AVMA) and Royal College of Veterinary Surgeons (RCVS), should be consulted to inform how such data is gathered, processed and governed. Ethical accountability also extends to the ownership and secondary use of uploaded videos, particularly when such data are repurposed for algorithm training, benchmarking, or shared with third parties, necessitating explicit consent and robust governance to ensure respect for user privacy and autonomy.

Looking to possible technical mitigation strategies, the current technical landscape provides various solutions which can aid in these considerations. Firstly, it is important the flow of data is clear in these scenarios, for example, a system for dog owners would capture a video on the user's device and then upload it to the cloud. The aim should be to restrict any personally identifiable information (PII) from reaching the cloud. This could be accomplished by the use of state-of-the-art segmentation algorithms such as Segment Anything (93) on device, performing a background replacement to show only the dog, as seen in popular video conferencing applications. This can be considered a catch-all solution, but could be made more robust by the use of on-device optical character recognition (OCR) for text detection and then blurring any detected text that may appear, ensuring that no residual PII is transmitted or persisted in back-end storage. These are only possible solutions, but must be reinforced with user-based responsibility. As such, introducing a user-driven video review prior to upload presents a second layer of protection whereby the user can scrub through the video for any information that they would not like uploaded to the cloud. Within this, the previously mentioned methods could also be available by allowing the user to select an object or area to blur. While user reviews provide an additional safeguard, primary ethical responsibility for preventing unintended disclosure of personal data rests with system designers and service providers, in line with privacy-by-design and data protection by default principles. Examples of this can be removing image and video metadata such as GPS and Device ID information.

### 4.4 Future research directions

A number of promising research avenues arise from the limitations identified. The introduction of ensemble and multi-module learning may compliment how gait is inherently multifaceted, especially considering the weakness of some of the present technologies such as occlusion. Given the common use of synthetic datasets, deeper research into synthetic-to-real domain adaptation, particularly shape adaption and real-world fine-tuning, could narrow the domain gap and provide more reliable models. Once parameter extraction is made more robust, the natural progression is toward diagnostic models for early lameness detection, post-operative recovery monitoring, degenerative disease progression, neurological vs. orthopedic classification, and even service-dog or sports-dog performance evaluation. Due to the variation of research domains in this area, we advocate for increased interdisciplinary collaboration to foster innovation and establish new standards in the field.

### 4.5 Broader significance of computer vision in canine and animal research

Computer vision has significant potential beyond the technical contributions outlined above. Unlike traditional methods like force plates or motion capture, vision-based systems can objectively observe the subject in their natural environment, providing longitudinal data unavailable through traditional means. This also reduces any false positives that may be indicated during examinations in clinics, hospitals or other areas foreign to the animal. Subtle gait changes often precede clinical symptoms. Automated systems could flag these issues earlier, reducing chronic pain and improving quality of life. This can be further expanded to monitor post-operative treatments or evaluate the effect of medication-based treatments. Together, a clear goal for improved animal welfare and early disease detection can be seen. Wider accessibility and the democratization of gait analysis is evident. Vision-based systems bypass the need for for specialized equipments and operators, allowing smaller clinics, research institutes and even dog owners to assess gait and health objectively. By increasing work into large-scale video datasets, reveal new breed-specific biomechanical patterns may be revealed, informing rehabilitation programs and risk assessments for orthopedic disorders. Lastly, advances in automated canine gait analysis may influence research fields focused on other quadrupeds, including bovine, wildlife and equine health analysis.

### 4.6 Conclusion

The synthesis of literature demonstrates that automatic canine gait analysis is not just an emerging domain, but a field that is on the edge of revolutionizing how veterinary biomechanics, rehabilitation and animal welfare can be approached. While current progress has been strongest in articulated pose and shape reconstruction and in kinematic parameter extraction, their significance lies in what they allow to now be possible. Vision-based methods offer a path toward objective, accessible, and longitudinal assessment of canine gait, capabilities that traditional methods such as pressure walkways and motion capture systems cannot provide outside specialized environments. Limitations such as imperfect monocular 3D reconstruction, inadequate dataset diversity, and the absence of frameworks that integrate both pose, gait parameters and clinical interpretations define not just obstacles, but a blueprint for maturing the field. Resolving these limitations provides a route for automated gait analysis in canines to transition from academic prototypes to clinically meaningful tools. In doing so, this technology has the potential to detect subtle changes in patients, support evidence-based decision-making for mobility and welfare, alongside democratizing gait assessments. It is clear that clinically validated 3D reconstruction and AI-based gait analysis has deep clinical applicability, but to bridge the gap between this and technical feasibility, a number of technical problems must be solved. Monocular 3D reconstruction methods require better occlusion handling, and either greater breed diversity within datasets or novel breed-agnostic methods, achieving an accuracy within the 10-20 mm joint-center localization bounds for soft tissue artifacts. From here, novel AI-based holistic gait analysis methods, which could utilize ensemble or multi-module machine learning frameworks, must be created. Specifically, these methods must be validated in partnership with clinicians to ensure that outputs are interpretable and clinically-relevant, enabling correlation with clinical outcomes and CMIs. Aligning future research with these identified requirements, the field can move toward reliable, accessible and clinically transformable systems that expand what clinicians, researchers and dog owners can understand about canine movement.
